# Supplementary material for: A Spinster-like Transporter at the Inner Membrane Complex is critical for Toxoplasma gondii cytokinesis, motility and invasion
Source: PLoS Pathog. 2026 Jan 28;22(1):e1013886. doi: 10.1371/journal.ppat.1013886 (PMC12871999; doi:10.1371/journal.ppat.1013886)
Supplement: Attachment — Submitted filename: Response to the Reviewers .pdf [file ppat.1013886.s018.pdf]

Answer to Reviewers – PLOS Pathogens

General statement

We thank the reviewers and the editor for their constructive and insightful comments, which have greatly improved the clarity and rigor of the manuscript. All comments have been addressed through additional experiments, text revisions, and figure updates. Changes are highlighted in the revised manuscript.

In addition to addressing the specific reviewers' comments, we have performed additional experiments to strengthen our conclusions. These experiments are included (**Fig. 4 and S5 Fig.**) and are discussed in the revised manuscript. We generated a new DCT1-mAID complemented cell line by introducing into the UPRT locus a construct containing the TgDCT1 N- and C-terminal regions flanking the PfDCT1 Spinster domain. Plaque assays showed that IAA-treated parasites fully restored lytic cycle progression and formed plaques comparable to the control strain. These results indicate that the *Plasmodium* and *Toxoplasma* transporters likely recognize the same substrate, consistent with both the functional complementation and the phylogenetic analysis presented in this study.

Reviewer 1

Part II – Major Issues: Key Experiments Required for Acceptance

The DCT1-mAID-HA line does not appear to localize to the daughter IMC […]. Can the authors confirm that it is degraded in the parental cells (as with DCT1-Ty above) and that it is associated with the alveoli using differential solubilization?

We have performed additional immunofluorescence analyses to confirm DCT1-mAID-HA localization and degradation, as well as the requested solubility assay. Consistent with previous observations, DCT1-mAID-HA is predominantly localized to the juxtanuclear region (likely the Golgi), but it is also detectable during daughter cell IMC formation when images are overexposed **(Fig. S4E).** IFAs further showed that the protein signal disappears once the mother cell is fully formed **(Fig. S4D).** Differential solubilization assays indicate that DCT1 behaves as an integral membrane protein associated with the alveoli **(Fig. S4F).**

The DCT1-mAID line efficiently degrades protein within 1 h, however from what I can see, all experiments were performed 24-30h post IAA addition. What are the first phenotypes seen upon DCT1 depletion? How is daughter cell budding affected at these early stages?

To examine early phenotypes upon DCT1 depletion, we performed a time-course analysis of DCT1-mAID parasites treated with IAA for 6, 12, 24, and 30 hours. Quantification of vacuoles containing dividing parasites showed a slight, non-significant increase at 6, 12, and 24 hours, with significant accumulation at 30 hours **(Fig. S6A).** Scoring division stages

revealed that after 12 hours of IAA treatment, parasites accumulated in the late stage of endodyogeny **(Fig. S6B),** indicating that daughter cell budding initiates normally but completion of cytokinesis is delayed. At later time points (24–30 hours), extensive morphological defects precluded precise stage assignment. Together, these results suggest that DCT1 is not required for the initiation of daughter cell formation but is important for progression and completion of endodyogeny.

Can the authors comment on the strong structural homology of DCT1 to HsSPNS2 could allow the utilisation of small molecules to more deeply probe the function of this transporter. Have the authors considered using FTY720-P (e.g. PMID: 21084291) or one of the small molecule inhibitors (e.g. PMID: 39820269) to modulate the role of the potential transporter and then determining the phenotype?

We thank the reviewer for this insightful suggestion, which helped improve the manuscript. To directly probe DCT1 function, we tested selective SPNS2 inhibitors, including compounds 11i (PMID: 38952222) and 33p (PMID: 38123824; 37010497). Host-cell toxicity assays showed that neither compound was cytotoxic after 24 h at 10 µM, whereas higher concentrations (25–50 µM) were toxic **(Fig. S10).** Accordingly, all parasite assays were performed at 10 µM. Under these non-cytotoxic conditions, both 11i and 33p induced IMC morphological defects **(Fig. 11A),** asynchronous division **(Fig. 11A–B),** and impaired intracellular replication **(Fig. 11C–D),** phenocopying aspects of DCT1 depletion while leaving DCT1 localization unchanged **(Fig. 11A).** Structural analysis indicated that the predicted conformation of 33p could plausibly fit within the TgDCT1 binding pocket **(Fig. S11C–D).** Attempts to obtain modelling files for 11i from the original authors were unsuccessful; instead, we compared amino acids predicted to interact with 11i in SPNS2 with the corresponding residues in TgDCT1. While most residues are not identical, several share similar physicochemical properties, suggesting that 11i could be accommodated within this pocket or bind to another region of DCT1. These results are now included in a new result section entitled: *SPNS2-targeting drugs impair T. gondii morphogenesis, intracellular replication, and synchronicity, mimicking DCT1 depletion*.

There is a fairly long section based on the phylogeny and alphafold models. As these data do not inform experiments in this manuscript, this section could be reduced.

The section describing the phylogenetic and AlphaFold analyses (previously in the Results and Methods) has been substantially shortened in the Results section. Excessive methodological details, including the full BLAST procedure, alignment and phylogenetic tree construction parameters, and the description of the MFS domain used for tree generation, have been removed. The revised Results section now focuses on the key phylogenetic relationships relevant to the study and provides a streamlined summary, in line with the reviewer's recommendation.

Part III – Minor Issues: Editorial and Data Presentation Modifications

Line 202- It is possible that this is due to processing, or to change in the 3’ UTR which can affect protein abundance.

Since the same 3′ UTR region was targeted to generate the KI-DCT1-Ty, DCT1-Ty-U1, and DCT1-mAID strains, the 3′ UTR modification is identical in all three lines and would be expected to similarly affect the proportion of the higher–molecular weight form of the protein in intracellular parasites. However, this is not observed. We therefore conclude that the mAID-3HA tag itself interferes with DCT1 processing and promotes its accumulation in the Golgi apparatus.

Line 447- The authors write: marker analysis revealed no defects in daughter budding upon DCT1 depletion, however, this result is not included in the text. Can this be included and quantified?

We have now addressed this reviewer’s concern. The results are described in the previous major comment section.

One potential way of determining the role of this transporter is to perform lipidomics at early timepoints, to look for changes. This would ideally be compared to a mutant with a similar phenotype which is not expected to change lipid composition. This however, is a potentially complex experiment, and so I offer it as a suggestion rather than a major comment.

We thank the reviewer for this suggestion. We performed untargeted lipidomic analyses on the DCT1-mAID strain treated with auxin for 12 hours, a time point chosen to precede the onset of severe morphological defects. These data indicate that DCT1-mAID parasites already display alterations in lipid composition **(Fig. S9A),** likely reflecting an early response to changes in DCT1 abundance at the daughter cell IMC. Notably, upon DCT1 depletion, we observed an accumulation of CerNDS and lysophospholipids compared with both TIR+IAA and DCT1-mAID −IAA controls **(Fig. S9B–D).** Together, these results provide insights into the role of DCT1 in maintaining lipid balance in the parasite. These data are now presented in a new Results section entitled: *Untargeted lipidomic analysis reveals DCT1-related alterations in parasite lipid balance.*

Reviewer 2

Part II – Major Issues: Key Experiments Required for Acceptance

The proteolytic processing of TgDCT1 from 86 kDa to 60 and 40 kDa species detected by a C terminal epitope tag suggests that the N-terminus is removed to create the active ~60 kDa species. It would be valuable to use purified tagged protein species of each size to determine N-terminal sequences by MS.

We thank the reviewer for this suggestion; however, due to the very low endogenous expression of TgDCT1, we were unable to purify enough of each fragment for N-terminal sequencing by mass spectrometry.

The authors could use a second copy knockdown system to probe the processing of TgDCT1 by creating truncation mutants. The transition in size to 60 kDa roughly corresponds to loss of the Nterminal fragment that lacks homology and the confidently predicted structure in alpha-fold. It could be interesting to follow the consequences of induced expression of a truncated copies of TgDCT1 in the mAID system to see if the N-terminal portion is required for correct localization to daughters and/or inhibition of activity. Also, given that Alphafold recognizes two types of domains in the transporter part of TgDCT1, I wonder if the second cleavage of 60 to 40 cuts these into separate parts.

We attempted to complement the DCT1-mAID mutant with an N-terminally truncated version of DCT1, fused to a Ty tag and expressed from the tub1 promoter at the UPRT locus. In the transfected population, we observed Ty-positive parasites with ER/Golgi localization; however, we were unable to obtain stable positive clones. We therefore did not include these data in the Results section but refer to them in the Discussion.

AlphaFold predictions identify a single MFS domain adopting the canonical architecture of two bundles of six transmembrane helices. Based on this prediction, we propose that the ~40 kDa species detected represents a degradation product rather than a biologically relevant processed form of the protein.

Given the nice analysis of sites that are conserved and distinct in human HsSPNS2 (Figure 9), it would be worth analyzing the defined missense mutations identified in other spinster proteins (human, Drosophila) to find some that are in conserved positions to introduce mutations for complementation analysis in the mAID 2-copy system described in the MS.

We attempted to generate a mutated version of the transporter by introducing the DCT1 R397A substitution, a mutation previously identified in several SPNS2 point-mutation studies as strongly impairing S1P transport. This construct was transfected into the DCT1-mAID background via integration at the UPRT locus. This mutant correctly located at the DC IMC, however, the proportion of positive parasites was too low to allow the isolation of stable clones. We are currently repeating this experiment and, if successful, the resulting data will be included in the revised manuscript.

With the caveat that the binding pocket is smaller in TgDCT1 (suggesting that it transports a distinct lipid), it would be an easy experiment to test whether treatment of Toxoplasma cultures with sphingosine 1-phosphate inhibitors (such as Fingolimod, ananalog) could be tested to see if they enhance or phenocopy the loss of TgDCT1.

Instead of targeting S1P receptors (e.g., fingolimod, which acts via functional antagonism of $S1P_1$ through receptor internalization), compounds such as 11i and 33p inhibit the S1P transporter SPNS2**.** Both 11i and 33p have been experimentally tested in wild-type parasites and phenocopy the loss of DCT1; these results are included in the manuscript (**Fig 11**) and detailed in the response to Reviewer 1.

Part III – Minor Issues: Editorial and Data Presentation Modifications

The authors describe using in silico methods to identify TgDCT1, but put the phylogenetic information, motifs and structure at the end where it seems like an afterthought. It would answer lots of questions that I had while reading the paper to move figure 8 to the front as figure 1. Figure 9 can stay where it is because it explores how TgDCT1 may function.

Thank you for this helpful suggestion. Figure 8 has been moved to the beginning of the manuscript as the new Figure 1. Supplementary figures have also been rearranged accordingly.

Given the strong likelihood that the non-conserved N-terminus is processed, it would be helpful to indicate in the Alphafold models and an alignment (Figure 8) where the "mature" protein would begin. Having this information in Figure 1 also helps the reader orient to the protein function. Also indicating the topology (C-terminus is cytoplasmic, allowing for the mAID system to work) in these models would be helpful.

Since the precise processing site of the non-conserved N-terminus has not been experimentally defined, we did not adjust the alignment to reposition the start of the mature protein. Instead, we indicate the N- and C-terminal regions directly on the TgDCT1 and PfDCT1 AlphaFold models (**Fig. 1D and S2B**), providing a clearer and less arbitrary representation of the protein topology.

Reviewer 3: Minor Revision

Part III – Minor Issues: Editorial and Data Presentation Modifications

No phase contrast or DIC images are included for immunofluorescence assays (IFAs) throughout the manuscript. Since visualization of the entire parasite morphology is important, the authors should include phase or DIC images alongside IFAs whenever possible.

Due to the large number of experiments and images, we have included DIC images for a representative example (GAP45/SAG1) to illustrate the alteration of parasite morphology (**S8B Fig.**). We have also included EM images of both DCT1-mAID and DCT1-Ty-U1 that provide a more informative and comprehensive view of the morphological defect (**Fig 5D and S6H)**

• 1A: Please specify that the Western blot was performed using intracellular parasite samples.

We have indicated that this western blot was performed using intracellular parasite samples (text, **Fig 2 legend**).

• 1B: In the Results section, clarify why the authors conclude that TgDCT1 expression occurs earlier than ISP1 based on the data shown.

We have detailed in the Results section, why TgDCT1 expression occurs earlier than ISP1. We also added colored arrows in the **Fig 2C** to highlight the presence of TgDCT1 in budding cells where ISP1 is absent. The figure legend and the text have been updated accordingly.

• 1D: The Results should explain the significance of using different solvents to determine TgDCT1's partitioning between pellet and supernatant fractions. Additionally, please explicitly state whether TgDCT1 behaves as a soluble or membrane-associated protein based on these results.

We have clarified the significance of using each solvent to assess TgDCT1 partitioning between pellet and supernatant fractions and have included a rationale explaining that the results support TgDCT1 as an integral protein of the alveolar membrane.

• 1D: A strong band at ~86 kDa is observed. Were these fractionation assays performed on extracellular or intracellular parasites? If extracellular, have the authors conducted IFAs on extracellular parasites to detect TgDCT1? Is there a signal observed under these conditions?

The solubility assays shown in **Fig. 2D** were performed on intracellular parasites. We also conducted IFAs on extracellular KI-DCT1-Ty tachyzoites, which revealed a punctate TgDCT1 signal near the nucleus (**S4A Fig.)**.

• 3C: Including phase or DIC images would help demonstrate that parasites are lingering near the ruptured vacuole.

While we appreciate the suggestion, we believe that including a phase-contrast or DIC image would not substantially improve clarity regarding the parasite's position relative to the disrupted vacuole. The GRA3 signal, along with other GRA markers, has been widely validated and extensively used to distinguish between intact and ruptured vacuoles.

• 4C: The legend or Results section should clarify the rationale for including panels of auxin-treated parasites.

The legend has been corrected to indicate that parasites were treated with rapamycin

• 4E: The authors need to better explain what they mean by "enlargement" of the basal complex.

The description to the enlargement of the basal complex has been better explained in the Results section.

• Including a marker for rhoptry bulbs would strengthen the interpretation of Figure 5.

We have now included a ROP5 staining in DCT1-mAID untreated or IAA-treated condition (**S7A Fig.**).

• Phase or DIC images would also be helpful here.

Although phase/DIC images are informative, we focused on IFAs as it provides the most direct assessment of DCT1 depletion effect on secretory and endosymbiotic organelles.

Based on IFAs shown in Figure 7, the authors state that TgDCT1 depletion affects maternal alveolar homeostasis (line 326). However, since TgDCT1 predominantly localizes to daughter cells and not the mother cell, the authors should provide additional discussion explaining how the observed defects in maternal alveolar homeostasis might arise.

We have now addressed this point in the Discussion. Although TgDCT1 predominantly localizes to daughter cells, we propose that the defects observed in maternal alveolar homeostasis result from the continuous accumulation of improperly transported components in the DC IMC. This explanation has been incorporated into the Discussion Section.

• Line 360: The authors do not provide direct experimental evidence that TgDCT1 functions in lipid transport. Therefore, they should significantly temper their conclusions regarding its role in lipid transport.

We have now tempered the conclusion on lipid transport in both the Results and Discussion sections. However the fact that inhibitors of the S1P transporter SPNS2, 11i and 33p, have been experimentally tested in wild-type parasites and phenocopy the loss of DCT1 provides supporting evidence. Additionally, we have introduced the R397A substitution in DCT1, a mutation previously shown in SPNS2 point-mutation studies to strongly impair S1P transport. Although the transgenic strain and associated data were not ready in time for this “minor revision”, we intend to include them in the final version of the paper if the results are conclusive.
